# Internet gaming disorder and its relationship with behavioral disorder and mother’s parenting styles in primary school students according to gender in Iran

**DOI:** 10.1186/s40359-021-00616-4

**Published:** 2021-07-26

**Authors:** Hossein Namdar Areshtanab, Fatemeh Fathollahpour, Mohammad Arshadi Bostanabad, Hossein Ebrahimi, Mina Hosseinzadeh, Marjaneh M. Fooladi

**Affiliations:** 1 Department of Mental Health and Psychiatric Nursing, Nursing and Midwifery Faculty, Tabriz University of Medical Sciences, Tabriz, Iran; 2 Department of Pediatric Nursing, Nursing and Midwifery Faculty, Tabriz University of Medical Sciences, Tabriz, Iran; 3 Department of Community Health Nursing, Nursing and Midwifery Faculty, Tabriz University of Medical Sciences, Tabriz, Iran; 4 University of Jordan, Amman, Jordan; 5 World Wide Nursing Service Network (WWNSN, PLLC), El Paso, TX USA

**Keywords:** Behavioral disorder, Internet gaming disorder, Parenting styles, Student

## Abstract

**Background:**

The problem of students’ gaming addiction has been related to the individual student’s characteristics and the influence of family environment. Researchers aimed to investigate if and how internet gaming disorder (IGD) of the elementary school boys and girls is related to behavioral disorder and their mother’s parenting style in Iran.

**Methods:**

This is a descriptive correlational study, involving 657 fifth and sixth-grade elementary school students in 2019. Researchers used a multistage stratified random sampling of students, their parents and their teachers. Data were collected using internet gaming disorder questionnaire (IGD 20), Rutter teacher behavioral disorder questionnaire and Baumrind Parenting Styles questionnaire (PSI). Obtained data were analyzed using SPSS version16 for descriptive statistics and Pearson correlation coefficient test.

**Results:**

Findings showed that IGD prevalence was 5.9% among primary school students with significant relationship between IGD and behavioral disorder among all participants (r = 0.23, *p* = 0.04); although it was insignificant among boys (r = 0.13, *p* = 0.11). Also the relationship between IGD and mother’s parenting style was significant in the total sample (r = 0.12, *p* = 0.03), in particular for girls and their mothers (r = 0.2, *p* = 0.001).

**Conclusions:**

The results of this study indicate the importance of family and parental involvement in prevention and management of IGD chiefly among girls. Healthcare professionals will benefit from knowing the problematic consequences of online gaming among school-age children and try to promote safe and healthy online behavior supported by a supervised family environment.

## Introduction

An increasing access to enticing computer games with exciting audiovisuals has captured the very young and school-age children. These games have replaced interest in television programs, physical exercise and interaction with peers. Children are gradually alienated from family and friends, with serious risk to their mental and physical health [1]. Others believe that computer games can provide opportunities for observational learning [2]. In studies focused on the effects of internet games on different parts of the brain have shown behavioral and cognitive changes such as improved orientation, increased attention span, improved visual and environmental attention [3]. The negative effects are listed as addiction to the game, increased anger and aggression, and other physical and psychological effects, where risks outweigh the benefits such as increased eye coordination among other positive aspects [4].

Many studies report that internet gaming provide emotional rewards alongside of aggressive behaviors during and after the game and gradually result in loss of sensitivity towards violence [4–6]. Persistent and recurrent use of the internet games, individually or with other players, lead to clinically significant impairment or distress. It remains to be seen if internet gaming disorder (IGD) is associated with compulsion, impulse control disorder, or gaming addiction [6]. A recent study has reported the prevalence of internet gaming disorder between 1%-10% in Europe and North America [7]. In a recent study in Iran, the prevalence of IGD among adolescents was at 5.3% [8]. In a large US study the observed IGD was up to five times more prevalent among the male children and diagnosed at 11.9% compared to 2.9% in female children [9]. In a recent systematic review only two studies reported no gender‐related difference in gaming addiction [10, 11].

Similar studies have shown a relationship between childhood behavioral disorders extended to adulthood mental disorders [12]. Behavioral disorders are common and disabling with impact on children's school performance [13]. Behavioral disorders are in a category of mental disorders characterized by uncommon persistent or repetitive behaviors in children of the same age who behave inappropriately, disrupt others, and interrupt activities. The three most common types of behavior disorders are attention deficit hyperactivity disorder, oppositional defiant disorder, and conduct disorder [12]. Researchers in Iran reported incidence of behavioral disorders in the whole country was 42.1% in children and adolescents with a higher rate among boys than girls [14].

A variety of factors are mentioned as predictors of IGD, among which are social and family environment. In a recent systematic review the potential role of “family and parenting factors” were highlighted as the only socially related category of risk or protective factors for IGD [15]. Dysfunctional parenting style is an important factor leading to the development of childhood personality and behavioral disorders [16]. Parenting style is defined as a set of parental attitudes and behaviors toward children and the way parents respond to their children’s emotions [17]. Parenting styles differs through various cultures, races, and economic groups. Families as social groups are influenced by the context around them and family relationships and parent–child interactions are each influenced by cultural context [18]. The methods that parents use to raise children has a major role in their children's mental health and later development of childhood behavioral problems such as negative interpersonal conflicts with others, family members and especially parents [19].

Parenting style is classified into three types based on responsiveness and demands as Authoritative, Authoritarian, and Permissive. Authoritative parents are sensitive to the child’s needs and their discipline method is supportive. Authoritarian parents tend to be harsh and apply hard punishment. Permissive parents are parent-centered and they seldom engage in child- rearing practices [18]. Children spent more time with their mothers and mothers’ parenting style have an essential effect on children’s behavioral disorder [20].

Behavioral patterns in early childhood and adolescence predict physical, mental, and social health outcomes in adulthood [12]. Similarly, behavioral problems of school-aged children regarding learning ability, communication skills and social interactions can influence their overall health later in life [21]. Moreover, behavioral disorders in childhood increase the risk of developing mental illness in adulthood [22].

The education system in Iran is divided into two main levels: primary (elementary) and secondary (high-school) education. All children spend six years in primary school starting at age six to 12 and attend high school from ages 12 to 18. Primary school education in Iran is mandatory by law.

Given the high popularity of internet games and their adverse effects on children’s overall health, and considering the paucity of published studies on this topic in Iran, researchers aimed to investigate the internet gaming disorders among the elementary school children, its gender differences and the relation between behavioral disorder and mother’s parenting style.

## Methods

### Study design & participants

In a cross-sectional and correlational survey study, researchers obtained data during February and March 2019 in Tabriz, Iran. Tabriz is one of the largest metropolitan cities located in northwestern part of Iran with five major urban districts. This study was conducted across all five distinct. The required sample size was calculated as 341 students for each gender (d = 0.1, SD = 0.94), based on Yen et al. [23], formula and a non-response rate of 10%. The final sample size of the study was determined as 682 with an equal number of 341 boys and 341 girls. Among the participants, 25 boys did not complete their questionnaires and excluded from the study, There was a drop rate of 7.33% for 25 male participants in our sampling.

Sampling method was a multistage stratified random selection by using a table to stratify the participants by schools, grades and classes. It is important to mention that in Iran most of the public and private schools are gender segregated, where boys and girls are taught separately by a same gender teacher. Initially one of the girl’s school and one of the boy’s school were randomly selected from each distinct. Later, at each school two classes were randomly chosen in each grade. Then, the IGD questionnaire was administered and completed by all students. After that, the behavioral disorder scale was completed by the teachers of selected classes. The parenting style questionnaire was completed by mothers for each selected student. The mean age of students was 11.72 ± 0.81, (age range of 10–13). There were 341 girls (51.9%) and 316 boys (48.1%). The average length of daily playing internet games was 103.35 ± 91.59 (5–600) minute for boys and 85.85 ± 76.56 (5–540) minute for girls. The majority of mothers were housewives (85.9% for boys and 85.6% for girls). The income status of most participants were middle class (60.4% for boys and 62.8% for girls).

The inclusion criteria for students were having no history of behavioral disorders reported by their teacher or parents enrolled in the study. Also, for teacher, we included those with at least one full semester of teaching at the selected school.

### Measures

The data collection tools included the IGD-20 questionnaire as the first standardized psychometric tool for assessing Internet Gaming Disorder (IGD) and according to the nine Internet Gaming Disorder (IGD) criteria recognized by the American Psychiatric Association and developed by Pontes [24]. The IGD questionnaire has twenty items with 9 IGD criteria listed in DSM-5 for evaluating online and offline gaming behaviors during a 12 months period. The IGD items are measured using a 5-point Likert scale with a minimum score of 1 [completely disagree] and a maximum score of 5 [completely agree]. The range of IGD scores are from 20 to 100 with a cut-off point of 71. Any score higher than 71 meets the criteria for IGD diagnostic case. A Farsi/Persian version of the questionnaire was recently validated in Iran with an internal consistency of 0.91 and a set test–retest reliability of 0.95; Moreover, using confirmatory factor analysis the construct validity of IGD was established [25]. In this study the face validity of questionnaires was evaluated by 10 faculty members at Tabriz University of Medical Sciences and the reliability of questionnaire was obtained through Cronbach's alpha of 0.88.

The second questionnaire used was Rutter teacher behavioral disorder, which has two forms as A (Parent Form) and B (Teacher Form). In this study, only B or teacher form with 30 questions was used to evaluate the behavioral disorder in children ages 7–13. This questionnaire consists of a three-point range that is respectively scored by 0 (never true), 1 (partly true) and 2 (totally true) [21]. The cut-off point of this form is 9. Rutter and his colleagues reported 0.89 for their tool reliability using the test–retest method. Validity of the Farsi/Persian version of questionnaire was reported as desirable and reliability was established as 0.90 by using test–retest. In a previous study in Iran, the construct validity of it was confirmed by using confirmatory and explanatory factor analysis [26]. The face validity of questionnaire was evaluated by 10 faculty members at Tabriz University of Medical Sciences and the reliability was 0.90 through Cronbach's alpha.

The third instrument we used was the Baumrind Parenting Styles Questionnaire (PSI). It is a famous questionnaire with 30 questions, scored on a 5-point Likert scale and three parenting styles sub-groups including authoritarian parenting styles assessed by questions# 1,6,10,13,14, 17,19,21,24,28, authoritative parenting styles by questions # 2,3,7,9,12,16,18,25,26,29 and permissive parenting styles by questions # 4,5,8,11,15,20,22,23,27,30 [27]. The reliability and content and construct validity of the tool was established in Iran and reported as desirable [28]. The face validity of questionnaires was evaluated by 10 faculty members at Tabriz University of Medical Sciences and reliability was obtained through Cronbach's alpha of 0.75.

### Data analysis

Collected data were analyzed by SPSS 16 software using the descriptive statistics (mean, standard deviation, frequency, and frequency percentage), and analytic statistics (Pearson correlation coefficient test and ANOVA), where *p* < 0.05 is considered statistically significant.

## Result

Table 1 represent the demographic and socio-economic characteristics of participants.

**Table 1:** Frequency distribution of the demographic and socio-economic characteristics of participants (total = 657)

Variable	Boys (N = 316)	Girls (N = 341)
Age (in years)		
M (SD)*	11.71 (0.803)	11.72 (0.815)
Occupation of mother N** (%)		
Housewife	256 (85.9)	285 (85.6)
Working	44 (4.7)	48 (14.4)
Playing duration (minute a day)		
M (SD)	85.85 (76.56)	103.35 (91.59)
Range	5–540	5–600
Internet game type N (%)		
Online game	12 (3.8%)	10 (2.9%)
Animal/food/fashion	0 (0%)	104 (30.6%)
Sport	66 (21%)	9 (2.6%)
Strategy	33 (10.5%)	7 (2.1)
Battle	43 (13.7%)	9 (2.6)
Puzzle	25 (7.9)	104 (30.6)
Driving	17 (5.4)	7 (2.1%)
More than three game items	119 (37.8)	90 (26.5%)
Family income status N (%)		
Income more than expense	19 (8.3)	31 (11.3%)
Income equal to expense	132 (60.4)	172 (62.8%)
Income less than expense	79 (31.3%)	71 (25.9%)

M* mean, SD standard deviation, N** number

Table 2 shows the frequency and Mean (SD) of IGD, behavioral disorder and parenting styles for students. Findings showed that 5.9% of students had gaming disorder (5.4% of girls and 6.5% of boys). About behavioral disorder findings showed that 40.8% of students had behavioral disorder with boys at a higher rate than girls (47.2% in boys and 34.9% of girls). The most common parenting style reported was authoritative at (92.8%) for boys (93.6%) for girls.

**Table 2:** Frequency and descriptive statistics of gaming disorder, behavioral disorder and parenting style among girls and boys

Total N (%)	Boys N (%)	Girls N (%)	Total N(%)	Boys Mean (SD)	Girls Mean (SD)	P value
Internet gaming disorder	22 (6.5)	17 (5.4)	39 (5.9)	49.57 (13.1)	47.56 (12.85)	T = 2.76, *p* = 0.1
Behavioral disorder	149 (47.2)	119 (34.9)	268 (40.8)	13.43 (13.31)	9.13 (10.30)	T = 4.60, *p* = 0.01
Parenting style						
Authoritarian	10 (4.5)	10 (3.6)	26.40 (6.1)	26.30 (5.98)	26.5 (6.27)	T = 1.60, *p* = 0.23
Authoritative	205 (92.8)	262 (93.6)	41.43 (5.9)	41.54 (5.71)	41.32 (6.13)	T = 2.60, *p* = 0.61
Permissive	6 (2.7)	8 (2.9)	24.73 (5.24)	24.73 (5.24)	24.7 (5.55)	T = 3.31, *p* = 0.54

Pearson correlation coefficient was used to determine significant differences or relationships between gaming disorder, behavioral disorder, and parenting style (Table 3). As findings show in Table 3 we found an significant relationship between IGD and behavioral disorder in our total sample (r = 0.23, *p* = 0.04); although this relationship was insignificant among boys (r = 0.13, *p* = 0.11). Meanwhile, the relationship between IGD and parenting style showed even more of an significant relationship in our total sample (r = 0.12, *p* = 0.03), and again a similar insignificant relationship for boys (r = 0.015, *p* = 0.82).

**Table 3:** Relationship between internet gaming with parenting styles and behavioral disorder

Sex	Boys		Girls		Total	
Variable	Gaming disorder		Gaming disorder		Gaming disorder	
Parenting styles						
Authoritarian	*p* = 0.13	r = − 0.01	*p* = 0.010	r = 0.15	*p* = 0.032	r = 0.15
Authoritative	*p* = 0.43	r = 0.05	*p* = 0.016	r = 0.14	*p* = 0.016	r = 0.14
Permissive	*p* = 0.83	r = − 0.14	*p* = 0.065	r = − 0.41	*p* = 0.065	r = − 0.11
Total	*p* = 0.82	r = 0.01	*p* = 0.001	r = 0.20	*p* = 0.03	r = 0.12
Behavioral disorder	*p* = 0.11	r = 0.13	*p* = 0.007	r = 0.44	*p* = 0.04	r = 0.23

As findings in Table 4 indicates there is a relationship between types of internet gaming and IGD and BD and this relationship revealed to be significant for girls (F = 6.07, *p* < 0.001, F = 2.06, *p* = 0.04). Also, for boys, we found a significant relationship just for the type of games they played and IGD (F = 2.76, *p* = 001, F = 1.82, *p* = 0.09). Students who played battle games showed a higher score for IGD and BD.

**Table 4:** The relationship between gaming disorder (IGD) and behavioral disorder (BD) with demographic and organizational characteristics

Variable	Boys (N = 316)	IGD	BD	Girls (N = 341)	IGD	BD
Age (in years)						
M (SD)	11.72 (0.815)	R = 0.32, *p* = 0.43	R = 0.2, *p* = 0.21	11.71 (0.803)	R = 0.28, *p* = 0.53	R = 0.32, *p* = 0.76
Playing duration (minute a day)	103.35 (91.59)	R = 0.75, *p* = 0.06	–	85.85 (76.56)	R = 0.43, *p* = 0.34	–
M(SD) Range	5–600		–	5–540		–
Game type N(%)						
Online game	12(3.8%)	F = 2.76*, *p* = 0.01 df = 6	F = 1.82 *p* = 0.093 Df = 6	10(2.9)	F = 6.07**, *p* < 0.01 Df = 7	F = 2.06 *p* = 0.04 Df = 5
Animal/food/fashion	0 (0%)			104 (30.6%)		
Sport	66 (21%)			9 (2.6)		
Strategy	33 (10.5%)			7 (2.1)		
Battle	43 (13.7%)			9 (2.6)		
Puzzle	25 (7.9)			104 (30.6)		
Driving	17 (5.4)			7 (2.1)		
More than three game items	119 (37.8%)			90 (26.5)		
Family income status N (%)						
Income more than expense	19 (8.3)	R = 0.41, *p* = 0.34	R = 0.31 *p* = 0.21	31 (11.3)	R = 0.32, *p* = 0.76	R = 0.12 *p* = 0.23
Income equal to expense	132 (60.4)			172 (62.8)		
Income less than expense	79 (31.3)			71 (25.9)		

*Post hoc P4,5 = 0.02, **post hoc P4,5 = 0.012

## Discussion

The present study revealed the prevalence of IGD among the Iranian primary school students was 5.9%, while previous studies in Iran found a 5.3% prevalence of IGD among the adolescents [8]. There has been a wide variety of prevalence IGD rates reported from other countries [12], and the variation may simply reflect the difference in culture and parenting styles. In a recent meta-analysis report, the prevalence of IGD among different population was found at 0.7–15.6% [7]. Perhaps increased rate of IGD is directly connected to the increased access to internet, smart phones, and other digital gaming devices most attractive to youth.

In this study, boys had a higher IGD mean scores and this finding is consistence with other recent studies [29, 30]. In an earlier study researchers found that males used the internet for entertainment and leisure activities [31]. Moreover, in a recent meta-analysis, males had a higher Internet gaming addiction compared to the female participants and among the studies reviewed, the smallest and largest effect sizes were found in the Asian regions. Other potential reason for gender differences may be related to girls being more supervised their family compared to boys due to the cultural fiber, where girls are further restricted and prevented from spending time on the internet.

In addition to gender, there was a significant relationship between the types of internet games and IGD prevalence, where students who played internet battle games showed a higher IGD scores in both boys and girls. In many cases, due to the young age, students could not differentiate between real and virtual worlds and subsequently showed the same violent behaviors played online in the real world.

The rate of behavioral disorder among primary school students in this study was 40.8%; with a higher rate among the boys than girls, showing statistical significance. In a recent study the rate of behavioral disorder among adolescent was reported between 15 and 44% [31], which is consistent with our findings. In another recent study the prevalence and severity of behavioral disorder were higher in boys than girls [32].

In this study male and female students reported the authoritarian parenting style as most prevalent, similar to other studies revealing the authoritative parenting style as the most dominant style [16, 33]. A strong authoritative parenting style in Iran shows a direct cultural component guided by religion and in this study questionnaire on parenting style was completed by mothers, which majority were homemakers (85.6%) with fulltime interaction with their children.

Researchers in this study found a significant relationship between internet gaming disorder, parenting styles, and behavioral disorder. This relationship was more significant among the girls compared to boys. Some of the studies have explored the relationship between IGD and adolescents’ mental health problems [34, 35]. In fact, one study identified a positive correlation between excessive use of internet gaming and an increased rate of depression, anxiety and psychotic behaviors among the adolescents [35]. Findings of another study suggests that gaming disorder can co-occur with a variety of other addictive behaviors [36]. It seems reasonable to explore a two-way communication between the variables and further investigate this recent phenomenon affecting our youth.

Romanian researchers examined the relationship between parenting style and IGD and found similar results as we did [37]. Similarly, Durkee et al. [38] found that students from 11 European countries (i.e., Austria, Estonia, France, Germany, Hungary, Ireland, Israel, Italy, Romania, Slovenia, and Spain) who did not live with both of their biological parents experienced low parental involvement, also known as parental unemployment and showed the highest relative risks for problematic internet use [38].

Our study limitations included the use of one form instead of both A and B of Rutter questionnaire of behavioral disorder. Therefore, our finding could have been limited for the adequate understanding of parents’ perspectives. Also, we only focused on the fifth and sixth-grade students in public schools. In order to generalize these results, it would be necessary to expand the sample further and include private or non-governmental schools and their student population using different academic approaches.

## Conclusion

Here we found the importance of family and mother’s involvement in prevention and management of IGD chiefly among girls. Healthcare professionals could benefit from knowing the problematic consequences of internet gaming and try to promote safe and healthy online behavior with counseling parents about effective parental involvement in a healthy family environment.

## Data Availability

The datasets used and/or analyzed during the current study are available from the corresponding author on reasonable request.
